# Immune-mediated thrombotic thrombocytopenic purpura in patients with and without systemic lupus erythematosus: a retrospective study

**DOI:** 10.1186/s13023-020-01510-9

**Published:** 2020-08-28

**Authors:** Cai Yue, Jian Su, Xiaohong Fan, Li Song, Wei Jiang, Jinghua Xia, Tao Shi, Xuan Zhang, Xuemei Li

**Affiliations:** 1 Department of Nephrology, Peking Union Medical College Hospital, Chinese Academy of Medical Science and Peking Union Medical College, 1 Shuai Fu Yuan, Beijing, 100005 China; 2 Thrombosis and Haemostasis Research Unit, Jiangsu Institute of Haematology, First Affiliated Hospital of Soochow University, 118 Shi Zi Jie, Suzhou, 215006 China; 3 Department of Emergency Medicine, Peking Union Medical College Hospital, Chinese Academy of Medical Science and Peking Union Medical College, 1 Shuai Fu Yuan, Beijing, 100005 China; 4 Department of Medical Intensive Care Unit, Peking Union Medical College Hospital, Chinese Academy of Medical Science and Peking Union Medical College, 1 Shuai Fu Yuan, Beijing, 100005 China; 5 Department of Rheumatology and Clinical Immunology, Peking Union Medical College Hospital, Chinese Academy of Medical Science and Peking Union Medical College, 1 Shuai Fu Yuan, Beijing, 100005 China; 6 Clinical Immunology Center, Chinese Academy of Medical Sciences and Peking Union Medical College, Beijing, 100730 China

**Keywords:** ADAMTS13 protein, Acquired thrombotic thrombocytopenic purpura, Systemic lupus erythematosus, Thrombotic microangiopathies

## Abstract

**Background:**

Thrombotic thrombocytopenic purpura (TTP) is associated with more deleterious outcomes in patients with systemic lupus erythematosus (SLE). However, ADAMTS13 (a disintegrin and metalloproteinase with a thrombospondin type 1 motif, member 13) levels and ADAMTS13 inhibitor were not routinely assayed in most previous studies. The objective of this study is to compare the characteristics and outcomes of immune-mediated TTP (iTTP) in patients with and without SLE.

**Methods:**

The medical data of 28 patients with iTTP from Peking Union Medical College Hospital were analysed. ADAMTS13 activity and ADAMTS13 inhibitor were measured in all patients.

**Results:**

All 28 patients had ADAMTS13 inhibitor and severe ADAMTS13 deficiency. iTTP was considered SLE-related (SLE-TTP) in 10 patients and primary (primary iTTP) in 18 patients. Renal involvement on presentation was more severe in patients with primary iTTP as determined by higher serum creatinine (162.7 ± 110.6 vs 73.3 ± 13.4 μmol/L, *p* < 0.01) and more prevalent acute kidney injury (72.2% vs 10.0%, *p* < 0.01) than in patients with SLE-TTP. More patients with SLE-TTP were treated with steroid pulse therapy (90.0% vs 16.7%, *p* < 0.01) and intravenous immunoglobulin (IVIG) (50.0% vs 5.6%, *p* = 0.01) compared to patients with primary iTTP. After adjustments for age and treatment, including steroid pulse therapy and IVIG treatment, the likelihood of clinical remission of SLE-TTP was significantly increased compared to that of primary iTTP (HR 7.6 [1.2, 50.1], *p* = 0.03). Mortality was also lower among patients with SLE-TTP than among patients with primary iTTP (0 vs 38.9%, *p* = 0.03).

**Conclusions:**

Renal involvement was less severe in patients with SLE-TTP than in patients with primary iTTP. The treatment responses and outcomes of SLE-TTP were no worse and perhaps even better than those of primary iTTP. When TTP is diagnosed in SLE patients, the ADAMTS13 level and ADAMTS13 inhibitor profile should be considered in addition to clinical features.

## Background

Thrombotic thrombocytopenic purpura (TTP) is an acute, life-threatening disorder characterized by microangiopathic haemolytic anaemia (MAHA), thrombocytopenia, and organ dysfunction. TTP results from innate or acquired deficiency of ADAMTS13 (a disintegrin-like and metalloproteinase with thrombospondin type 1 motif 13), a protease that cleaves von Willebrand factor multimers, the deficiency of which results in unusually large von Willebrand factor multimers and a risk of platelet thrombi in small vessels with high shear rates [1]. Acquired TTP, also known as immune-mediated TTP (iTTP), results from autoantibodies against ADAMTS13. iTTP can be primary (primary iTTP) when no obvious underlying precipitating cause/disease is present or accompany other conditions, especially autoimmune diseases [2, 3].

Systemic lupus erythematosus (SLE) is a systemic autoimmune disease with multisystem involvement and is among the most common causes of iTTP, while TTP is a rare and severe complication of SLE [4]. The traditional estimate of TTP among lupus patients varies between 1% and 4% according to different diagnostic criteria [5]. TTP is associated with high mortality and severe end-organ damage among lupus patients [6–9]. Some authors reported more deleterious outcomes in patients with SLE-associated TTP (SLE-TTP) compared to patients with primary iTTP despite more aggressive treatment in these patients [10].

However, in most previous studies, ADAMTS13 levels and ADAMTS13 inhibitor were not assessed, and the diagnosis of TTP was based on clinical “pentads” of fever, thrombocytopenia, MAHA, and neurological and/or renal impairment, which may be misleading since considerable overlap exists between TTP and SLE regarding presenting features, and causes of thrombotic microangiopathy (TMA) may vary among lupus patients [11]. The characteristics and optimal treatment of patients with SLE-TTP remain unclear, and opinions about SLE-TTP should be updated with insight into ADAMTS13 activity. The aim of this study was to compare the clinical characteristics, treatment responses, and outcomes of patients with SLE-TTP and primary iTTP with evaluation of their ADAMTS13 activities.

## Results

### Baseline demographics

**Fig. 1:**
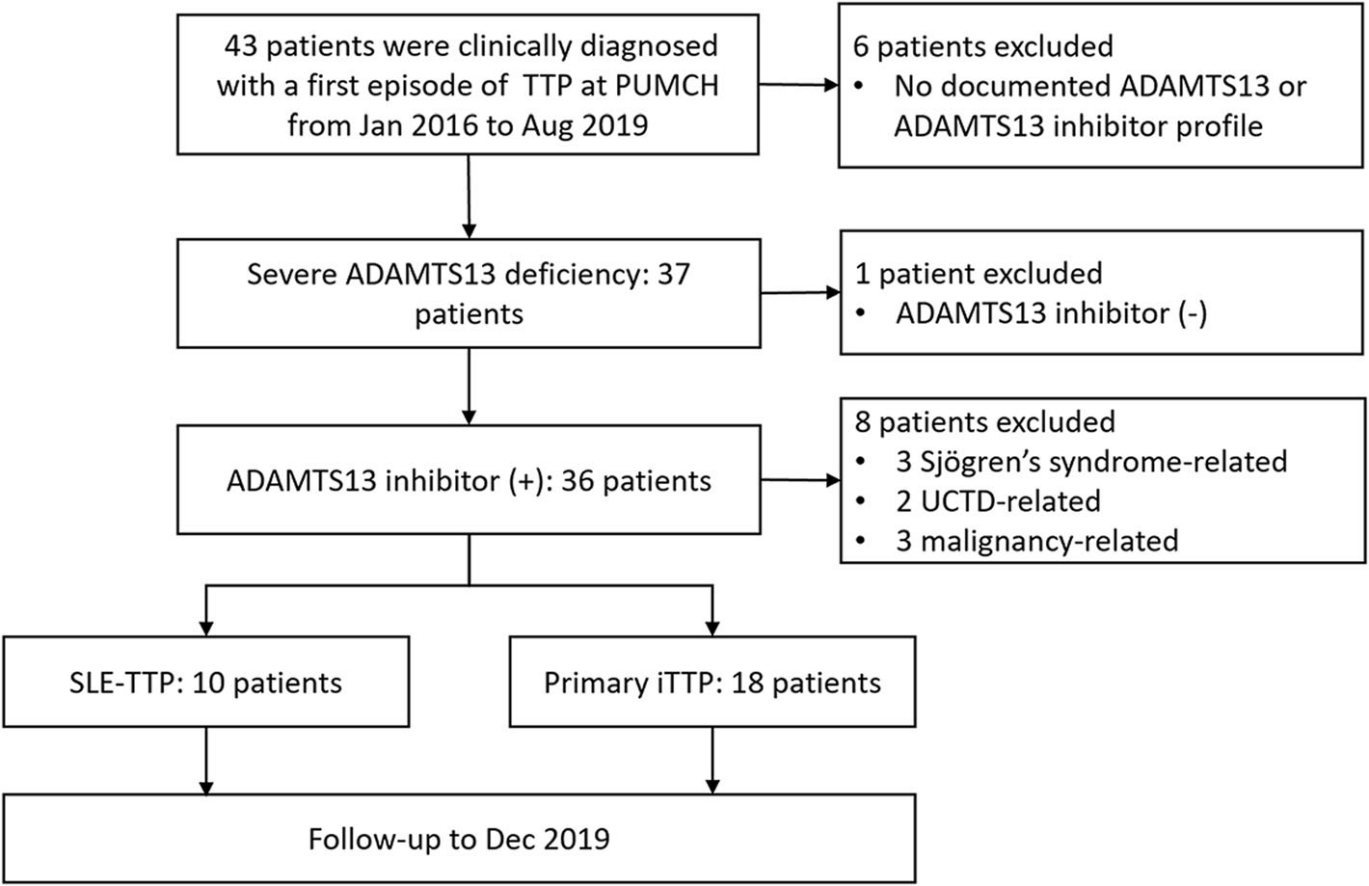
Flow chart of patient selection. Abbreviations: iTTP, immune-mediated TTP; UCTD, undifferentiated connective tissue disease; SLE, systemic erythematosus; SLE-TTP, SLE-associated TTP; TTP, thrombotic thrombocytopenic purpura

From Jan. 2016 to Aug. 2019, 43 hospitalized patients were clinically diagnosed with their first episode of TTP at Peking Union Medical College Hospital (PUMCH). ADAMTS13 activity and ADAMTS13 inhibitor were available in 37 patients. All 37 patients had severe ADAMTS13 deficiency, and ADAMTS13 inhibitor was detected in 36 patients. Of the 36 patients with acquired ADAMTS13 deficiency, TTP was considered SLE-related in 10, Sjögren’s syndrome-related in three, undifferentiated connective tissue disease (UCTD)-related in two, malignancy-related in three, and primary in 18 patients (Fig. 1).

The baseline characteristics of the 10 patients with SLE-TTP and the 18 patients with primary iTTP are listed in Table 1. No patients had a previous history of TTP. All patients with SLE-TTP were female, while a male predominance was observed in patients with primary iTTP (female ratio 100% vs 38.9%, *p* < 0.01). Patients with primary iTTP were older compared to patients with SLE-TTP (48.6 ± 10.8 vs 32.6 ± 16.9 years, *p* = 0.02). All patients had no comorbidity of clinical significance, except for one patient with primary iTTP who had histories of hypertension and diabetes and a remote history of colon cancer that had been surgically resected.

**Table 1:** Baseline characteristics of patients with immune-mediated thrombotic thrombocytopenic purpura.

	Primary iTTP (*n* = 18)^a^	SLE-TTP (*n* = 10)^a^	*p* values
Demographic data			
Age, yr	48.6 ± 10.8	32.6 ± 16.9	**0.02**
Female, *n*	7 (38.9)	10 (100.0)	**< 0.01**
New-onset TTP, *n*	18 (100.0)	10 (100.0)	1.00
Comorbidities	1 (5.6)	0 (0.0)	1.0
Haematological abnormalities			
Platelet count, × 10^9^/L	17.4 ± 14.4	14.7 ± 12.5	0.62
Haemoglobin, g/L	83.5 ± 23.3	78.8 ± 16.6	0.58
Lactate dehydrogenase, U/L	2015.5 ± 2016.9	1272.4 ± 652.1	0.27
Severe ADAMTS13 deficiency	18 (100.0)	10 (100.0)	1.00
ADAMTS13 inhibitor	18 (100.0)	10 (100.0)	1.00
Immunological parameters			
Complement C3 (g/L)	0.88 ± 0.24	0.76 ± 0.16	0.09
Complement C4 (g/L)	0.17 ± 0.07	0.12 ± 0.08	0.14
ANA	5 (27.8)	10 (100.0)	**< 0.01**
Low titre (< 1:320)	4 (22.2)	3 (30.0)	0.67
High titre (≥1:320)	1 (5.6)	7 (70.0)	**< 0.01**
Anti-dsDNA	0 (0.0)	4 (40.0)	**0.01**
Anti-SSA	4 (22.2)	7 (70.0)	**0.02**
Anti-SSB	0 (0.0)	2 (20.0)	0.12
Anti-U1RNP	0 (0.0)	2 (20.0)	0.12
Anti-Sm	0 (0.0)	1 (10.0)	0.36
APLs	1 (5.6)	1 (10.0)	1.00
SLEDAI	–	20.5 ± 7.7	–

*Abbreviations*: *ANA* anti-nuclear antibody, *APLs* anti-phospholipid antibodies, *CNS* central nervous system, *dsDNA* double-stranded DNA, *iTTP* immune-mediated TTP, *SLE* systemic lupus erythematosus, *SLEDAI* SLE disease activity index, *SLE-TTP* SLE-associated TTP, *TTP* thrombotic thrombocytopenic purpura

^a^ Values are expressed as the means ± SDs or the medians (interquartile ranges). Attributes values are expressed as numbers (percentages).

### Haematological abnormalities

All patients had severe ADAMTS13 deficiency and were positive for serum ADAMTS13 inhibitor. Platelet counts, lactate dehydrogenase (LDH) levels and haemoglobin levels on presentation were similar between the two groups (Table 1).

### Immunological parameters

Among the patients with primary iTTP, 27.8% were positive for anti-nuclear antibody (ANA), but the titres were low. Among the patients with SLE, 40% were positive for anti-dsDNA antibody, and 70% were positive for anti-SSA antibody. The average SLE disease activity index (SLEDAI) was 20.5 ± 7.7 (Table 1).

### Organ involvement

Organ involvement among patients with iTTP is described in Table 2. All patients in both groups had CNS involvement, and 64.3% had significantly elevated troponin I (cTnI). No significant differences in the prevalence and severity of CNS and cardiac involvement were observed between the two groups.

**Table 2:** Organ involvement of patients with immune-mediated thrombotic thrombocytopenic purpura.

	primary iTTP (*n* = 18)^a^	SLE-TTP (*n* = 10)^a^	*p* values
CNS involvement, n	18 (100.0)	10 (100.0)	1.00
Severe	17 (94.4)	10 (100.0)	1.00
Seizure	13 (72.2)	4 (40.0)	0.12
Coma	15 (83.3)	7 (70.0)	0.63
Stroke	3 (16.7)	3 (30.0)	0.63
Psychosis	0 (0.0)	2 (20.0)	0.12
Renal involvement			
Serum creatinine, μmol/L	162.7 ± 110.6	73.3 ± 13.4	**< 0.01**
BUN, μmol/L	15.4 ± 10.1	7.9 ± 2.7	**< 0.01**
AKI	13 (72.2)	1 (10.0)	**< 0.01**
Stage 1	5 (27.8)	1 (10.0)	0.37
Stage 2	6 (33.3)	0 (0.0)	0.06
Stage 3	2 (11.1)	0 (0.0)	0.52
Proteinuria level, dipstick			
Negative	2 (11.1)	1 (10.0)	1.00
Mild (± to +)	7 (38.9)	6 (60.0)	0.43
Moderate (++)	9 (50.0)	2 (20.0)	0.22
Large (+++)	0 (0.0)	1 (10.0)	0.36
Cardiac involvement			
cTnI (μg/L)	0.47 (0.11, 1.00)	0.10 (0.05, 0.64)	0.36
cTnI> 0.25 μg/L	13 (72.2)	5 (50.0)	0.41
MAP, mmHg	85.3 ± 11.9	94.6 ± 13.9	0.08

*Abbreviations*: *AKI* acute kidney injury, *BUN* blood urea nitrogen, *CNS* central nervous system, *cTnI* troponin I, *iTTP* immune-mediated TTP, *MAP* mean arterial pressure, *SLE* systemic lupus erythematosus, *SLE-TTP* SLE-associated TTP, *TTP* thrombotic thrombocytopenic purpura

^a^ Values are expressed as the means ± SDs or the medians (interquartile ranges). Attributes values are expressed as numbers (percentages)

Notably, renal impairment was more severe in patients with primary iTTP than in patients with SLE-TTP. Serum creatine in the patients with primary iTTP was significantly higher than that in the patients with SLE-TTP (162.7 ± 110.6 vs 73.3 ± 13.4 μmol/L, *p* < 0.01). More patients in the primary iTTP group had acute kidney injury (AKI) compared to the SLE-TTP group (72.2% vs 10.0, *p* < 0.01). However, severe AKI (stage 3) was rare.

### Treatment

The treatments and outcomes of the 18 patients with primary iTTP and the 10 patients with SLE-TTP are listed in Table 3. No significant difference in the number of plasma exchange (PE) sessions needed for remission was found between the two groups. However, other treatments were quite different between the two groups. More patients with SLE-TTP received steroid pulse therapy (90.0% vs 16.7%, *p* < 0.01), intravenous immunoglobulin (IVIG) (50.0% vs 5.6%, *p* = 0.01), and immunosuppressants (90.0% vs 5.6%, *p* < 0.01) than patients with primary iTTP, while rituximab was the mainstay of treatment in patients with primary iTTP (55.6%).

**Table 3:** Treatment and outcomes of patients with immune-mediated thrombotic thrombocytopenic purpura.

	primary iTTP (*n* = 18)^a^	SLE-TTP (*n* = 10)^a^	Hazard ratio SLE-TTP/primary iTTP	*p* values
Therapies				
PE, *n*	17 (94.4)	7 (70.0)		0.12
Number of PE sessions needed for clinical remission, *n*	10.0 (7.0, 15.0)^b^	8.0 (0, 14.8)		1.00
High-dose steroids, *n*	18 (100.0)	10 (100.0)		1.00
Steroid pulse therapy, *n*	3 (16.7)	9 (90.0)		**< 0.01**
Immunosuppressants, *n*	1 (5.6)	9 (90.0)		**< 0.01**
CYC, *n*	1 (5.6)	8 (80.0)		**< 0.01**
MMF, *n*	0 (0.0)	1 (10.0)		0.37
Rituximab, *n*	10 (55.6)	2 (20.0)		0.11
IVIG, *n*	1 (5.6)	5 (50.0)		**0.01**
Therapeutic responses				
Follow-up, months	12.8 (0.3, 34.6)	32.7 (14.2, 43.0)		0.14
Clinical response	11 (61.1)	10 (100.0)	6.4 [1.3, 30.9] ^d^	**0.02**^d^
Clinical remission	11 (61.1)	10 (100.0)	7.6 [1.2, 50.1] ^d^	**0.03**^d^
Exacerbation	2 (18.2)^b^	2 (20.0)		1.00
Refractory	7 (46.7) ^c^	2 (20.0)		0.23
Death	7 (38.9)	0 (0.0)		**0.03**

*Abbreviations*: *CYC* cyclophosphamide, *iTTP* immune-mediated TTP, *IVIG* intravenous immunoglobulin, *MMF* mycophenolate mofetil, *SLE* systemic lupus erythematosus, *SLE-TTP* SLE-associated TTP, *PE* plasma exchange, *TTP* thrombotic thrombocytopenic purpura

^a^ Values are expressed as the means ± SDs or the medians (interquartile ranges). Attributes values are expressed as numbers (percentages).

^b^ Patients who died during hospitalization and did not achieve haematological remission were not included in the analysis.

^c^ Three patients who died before completing five sessions of TPE were not included in the analysis.

^d^ HR and *p* values are adjusted for age and treatment, including steroid pulse therapy and IVIG treatment.

### Response to treatment

**Fig. 2:**
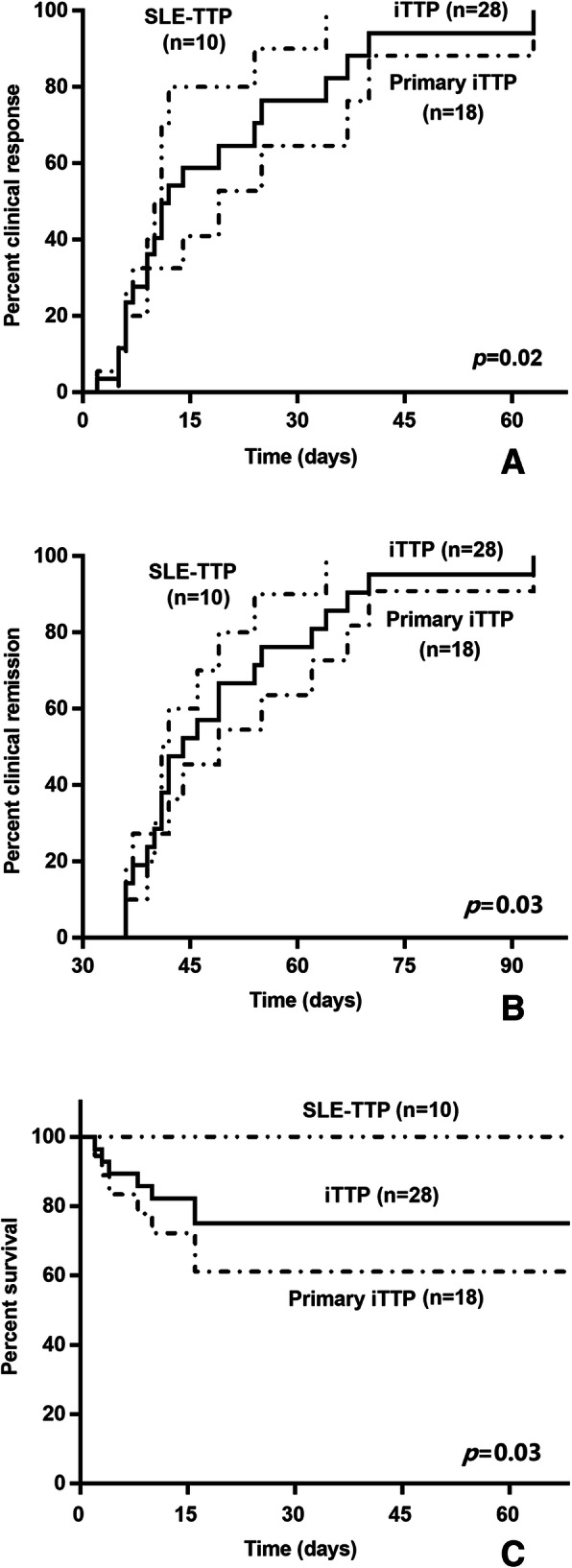
Outcomes of patients with immune-mediated thrombotic thrombocytopenic purpura. **a**. Cumulative incidence of a clinical response. **b**. Cumulative incidence of clinical remission. **c**. Cumulative incidence of survival. iTTP, immune-mediated TTP; SLE, systemic lupus erythematosus; TTP, thrombotic thrombocytopenic purpura

The overall mortality rate was 25.0%. Death was tentatively attributed to heart failure (*n* = 3), infection (*n* = 2), cerebral haemorrhage (*n* = 1), and multiorgan failure (n = 1). All deaths occurred in the primary iTTP group. Mortality was significantly lower among patients with SLE-TTP than among patients with primary iTTP (0 vs 38.9%, *p* = 0.03). All of the remaining patients achieved clinical remission. The median intervals from TTP diagnosis to clinical remission were 45.2 ± 8.4 days for patients with SLE-TTP and 53.7 ± 17.9 days for patients with primary iTTP. After adjustments for age and treatment, including steroid pulse therapy and IVIG treatment, the chances of a clinical response and clinical remission of SLE-TTP were significantly increased compared to those for primary iTTP (HR 6.4 [1.3, 30.9], *p* = 0.02, and 7.6 [1.2, 50.1], *p* = 0.03, respectively) (Fig. 2). During a median follow-up of 20.8 (0.9, 41.3) months, one recurrence was observed in a patient with primary iTTP at 16 months after discharge, and no recurrence was observed in patients with SLE-TTP.

## Discussion

SLE is one of the most common causes of iTTP [4]. SLE-TTP and primary iTTP might be overlapping disease entities. Up to 71% of patients with TTP have been reported to present with positive anti-nuclear antibody, and the rate of subsequent SLE development among patients with primary iTTP ranged between 7.1 to 11.1% [12–14]. Despite the substantial overlap and improving understanding of primary iTTP, the clinical characteristics and optimal treatment of patients with SLE-TTP remain unclear.

SLE-TTP has been reported to be associated with a high mortality rate and a poor response to treatment [6–9]. Kwok reported an in-hospital mortality rate of 46.1% among 26 patients with SLE-TTP, while Merayo-Chalico et al reported a long-term mortality rate of 20% among 22 patients [6, 8]. However, these studies did not systematically include ADAMTS13 activity in the definition of TTP. Jiang et al reviewed case reports on SLE-TTP from 1999 to 2011. ADAMTS13 levels were available in 32/105 patients, while only 13/32 of these patients had severe ADAMTS13 deficiency, indicating that in most of previous studies, the diagnosis of SLE-TTP was not based on ADAMTS13 levels, and that a substantial proportion of lupus patients with TMA may be diagnosed with TTP despite non-severe ADAMTS13 deficiency [9]. Other forms of TMA apart from TTP might have been included in these studies, confounding the results. Few studies have compared SLE-TTP with primary TTP. Letchumanan et al compared 10 patients with idiopathic TTP and eight patients with SLE-TTP and reported higher mortality and slower remission in the patients with SLE-TTP than in the patients idiopathic TTP [10]. Again, TTP was diagnosed based on the Moschowitz clinical pentads, and ADAMTS13 activity and ADAMTS13 inhibitor profiles were lacking.

In this study, all patients were confirmed to have severe ADAMTS13 deficiency and the presence of ADAMTS13 inhibitor. Interestingly, when severe acquired ADAMTS13 deficiency was considered in the diagnosis of TTP, the clinical picture of SLE-TTP was quite different from previous descriptions. Compared to patients with primary iTTP, patients with SLE-TTP had less severe renal involvement, better treatment responses and lower mortality. However, several limitations must be discussed.

First, whether the less severe organ involvement and better outcomes of SLE-TTP observed in our study represented the divergent underlying pathogenesis of SLE-TTP and primary iTTP or were attributable to the different baseline characteristics and treatment strategies between the two groups is not clear.

In our study, patients with SLE-TTP were significantly younger than patients with primary iTTP, and a female predominance was evident. In a predictive model developed by the French TMA Reference Center, age was a strong predictor of death due to primary iTTP. The worse prognosis of TTP in older patients was proposed to be attributable to comorbidities, including hypertension, resulting in chronic endothelial and vascular dysfunction, as well as age-associated loss of vascular compliance, which might result in higher shear stress and more severe vascular wall constraints and organ injury during TTP [15]. The younger age of the patients with SLE-TTP might have contributed to the better prognosis and less severe renal involvement of these patients compared to the patients with primary iTTP.

The treatment strategies were also significantly different between the patients with SLE-TTP and primary iTTP. More patients with SLE-TTP were treated with steroid pulse therapy and IVIG compared to patients with primary iTTP. Steroids are considered an adjunct to PE in the treatment of iTTP [16, 17]. Evidence supporting steroid pulse therapy in patients with primary iTTP is limited. A randomized clinical trial showed that corticosteroid pulse therapy may decrease mortality and increase the rate of complete remission among patients with clinically diagnosed TTP [18]. Many authors agree that for severe cases of TTP, steroid pulse therapy should be considered [19, 20]. Considering the role of autoimmunity in both SLE-TTP and primary iTTP and the overlap of these two disease entities, primary iTTP may benefit from corticosteroid pulse therapy.

However, in our Cox proportional hazards regression, coexisting SLE was associated with a better treatment response independent of age and treatment, reflecting the differing underlying pathogenesis mechanisms of SLE-TTP and primary iTTP. The mechanism involved in the loss of tolerance and subsequent development of anti-ADAMTS13 antibodies in patients affected by acquired TTP is not clear, but both genetic and environmental factors have been postulated to contribute to the development of primary iTTP [21]. In SLE-TTP, inflammatory conditions in active SLE might function as triggers for the generation of ADAMTS13 autoantibodies. Inflammatory conditions may affect host glycosylation by upregulating glycosyltransferase activities that modify the glycan structure of glycoproteins, and carbohydrate modifications modulate the immune recognition of allergens by epithelial and dendritic cells [22, 23]. ADAMTS13 is a highly glycosylated protein [24]. Inflammatory conditions in active SLE might result in hyperglycosylation or deglycosylation of ADAMTS13, resulting in higher immunogenicity by these molecular changes and contributing to the onset of acquired TTP.

Second, since PUMCH is a tertiary referral centre in China, this cohort may have represented a more severe subset of patients or patients at a later disease stage among all patients with iTTP. Our mortality rate was higher than the mortality rate of 4.3–12.8% reported by several large TTP cohorts [15, 25, 26]. However, our patients also had more severe end-organ damage on presentation, including more prevalent myocardium involvement and CNS involvement, both of which are strong prognostic factors of iTTP [15, 27]. Specifically, the fraction of patients with prominent troponin I (cTnI) elevation (> 0.25 μg/L) in our cohort was higher than that reported in a subset of the French TMA registry cohort (64.2% vs 41.3%), while mortality was similar between the cohorts (25.0% vs 24.8%) [27]. The fraction of patients with severe CNS involvement was higher than that reported by the Oklahoma registry (96.4% vs 52.6%) [25]. AKI was also prevalent, and the rate of AKI was similar to that reported in a French cohort of ICU patients (50.0% vs 58.7%) [26]. Thus, our cohort might have represented a subset of patients with more severe end-organ damage and therefore worse outcomes.

Ultimately, the sample size was small, which may limit the strength of the results. However, although iTTP may accompany all kinds of autoimmune diseases, considering the rarity of iTTP, a large sample size would be difficult to achieve [3]. Moreover, for most facilities, including ours, ADAMTS13 activity and ADAMTS13 inhibitor assays became routine in clinical practice only in recent years. Time is still required for physicians to gain experience in managing patients with different forms of secondary iTTP. Further observations with more patients are needed to verify our findings.

## Conclusions

Renal involvement was less severe in patients with SLE-TTP than in patients with primary iTTP. The treatment responses and outcomes of SLE-TTP were no worse and perhaps even better than those of primary iTTP. When TTP is diagnosed in SLE patients, the ADAMTS13 level and ADAMTS13 inhibitor profile should be considered in addition to clinical features.

## Methods

### Aim and design

To compare the characteristics and outcomes of iTTP in patients with and without SLE, patient records were retrospectively reviewed at PUMCH.

### Patient selection

The eligibility criteria for the study included: 1) MAHA, thrombocytopenia, and end-organ injury, including renal involvement or CNS involvement; 2) severe ADAMTS13 deficiency; and 3) positive serum ADAMTS13 inhibitor. SLE was diagnosed based on the revised 1997 American College of Rheumatology (ACR) diagnostic criteria [28]. The diagnosis of SLE was further confirmed with the 2019 EULAR/ACR classification criteria [29].

Patients with comorbidities that may result in acquired ADAMTS13 deficiency other than SLE, including other autoimmune diseases, malignancy, and systemic infection, were excluded [16].

### Blood sampling

Blood samples were collected upon clinical suspicion of TTP and before therapeutic apheresis (TPA) or plasma infusion.

### ADAMTS13 activity and ADAMTS13 inhibitor assays

ADAMTS13 activity and ADAMTS13 inhibitor were assayed as previously described by evaluating collagen-binding activity (CBA) at the Thrombosis and Haemostasis Research Unit, First Affiliated Hospital of Soochow University [11]. In brief, plasma samples from patients and normal individuals were placed in Slide-A-Lyzer mini-dialysis units (Pierce, USA) and immersed in pre-warmed dialysis buffer consisting of 5 mmol/L Tris-HCl, 0.1% Tween 20, and 1.5 mol/L urea, with a pH of 8.3. Dialysis was performed at 37 °C for 12 h. An equal volume of the same sample was removed before dialysis and kept at room temperature during dialysis as a control. The collagen type III binding capacities of the samples were then detected by enzyme-linked immunosorbent assay (ELISA). The data were analysed as the fraction of CBA remaining after dialysis compared with the CBA in the individuals’ baseline samples. One hundred percent minus the residual CBA (R-CBA) was regarded as the ADAMTS13 activity. The resulting ADAMTS13 activity detected was either 100% or 0% due to the extended dialysis period. ADAMTS13 deficiency was considered severe in patients with undetectable enzymatic activity.

For the ADAMTS13 inhibitor assay, patient plasma was incubated with normal human plasma (9:1) at 37 °C for 3 h, and the residual ADAMTS13 activity was measured. ADAMTS13 inhibitor was considered positive if the residual ADAMTS13 activity was 0% and negative if the residual ADAMTS13 activity was 100%.

### Treatment

Daily PE of one plasma volume replaced with fresh frozen plasma was started immediately following clinical suspicion of TTP and continued until platelet counts exceeding 150 × 10^9^/L were measured over two consecutive days. High-dose corticosteroid therapy, defined as prednisone 1 mg/kg/day or an equivalent dose of methylprednisolone, was applied to all patients. For severe cases, steroid pulse therapy, defined as administration of intravenous methylprednisolone 1 g/day for 3 consecutive days, followed by high-dose steroid therapy (1 mg of oral prednisone/kg/day), and IVIG (Rongsheng, Chengdu, China) 1 g/kg given in three or five divided doses immediately after PE were considered. For refractory cases or cases with exacerbation, the anti-CD20 monoantibody rituximab was considered. The dose of rituximab was 375 mg/m^2^ intravenously once weekly for four consecutive weeks and immediately after PE.

### Data collection

The clinical, serologic and medication data of the patients with TTP due to severe acquired ADAMTS13 deficiency were collected. Baseline demographic data were collected at the time of admission. Patients were followed up on a regular basis depending on their clinical status after discharge.

### Definitions and outcomes

iTTP was defined as TMA characterized by severe ADAMTS13 deficiency due to an inhibitor directed against ADAMTS13. Primary iTTP was defined as iTTP without comorbidities that may cause acquired ADAMTS13 deficiency [1]. SLE-TTP was defined as iTTP in patients with SLE. The SLEDAI was scored for each patient with SLE at the time of admission according to the SLEDAI-2000 index [30]. AKI was defined and staged according to the 2012 Kidney Disease: Improving Global Outcomes (KDIGO) clinical practice guideline for AKI [31]. The mean arterial pressure (MAP) was calculated with systolic blood pressure and diastolic blood pressure as previously described [32]. Clinical response was defined as sustained normalization of platelet counts above 150 × 10^9^/L and LDH after cessation of PE. Clinical remission was defined as a clinical response after cessation of PE maintained for > 30 days. Exacerbation was defined as a reduction in the platelet count to below 150 × 10^9^/L, an increased LDH level, and the need to restart PE within 30 days of the last PE after a clinical response to PE. Refractory disease was defined as a lack of a sustained platelet count increase or platelet counts < 50 × 10^9^/L and a persistently elevated LDH level despite five PEs and steroid treatment [2].

### Statistical analysis

Quantitative variables are reported as the mean ± standard deviation or the median and interquartile range. Categorical variables were compared using Fisher’s exact test. Differences in the mean or median values between defined patient groups were compared using Student’s t-test or the nonparametric Mann–Whitney U test. To evaluate the influence of co-existing SLE on patients’ outcomes, Cox proportional hazards regression was used with adjustments for age and treatment, including steroid pulse therapy and IVIG, with the time of presentation as the start of the follow-up. All statistical tests were two-sided, with significance defined as *p* < 0.05. All statistical analyses were performed using SPSS software, version 22.0 (IBM, USA).

## Data Availability

The datasets used and/or analysed during the current study are available from the corresponding author upon reasonable request.

## Abbreviations

ACR: American College of Rheumatology
ADAMTS13: A disintegrin and metalloproteinase with a thrombospondin type 1 motif, member 13
AKI: Acute kidney injury
ANA: Anti-nuclear antibody
iTTP: Immune-mediated thrombotic thrombocytopenic purpura
CBA: Collagen-binding activity
CNS: Central nervous system
CR: Complete remission
cTnI: Troponin I
ELISA: Enzyme-linked immunosorbent assay
HR: Hazard ratio
IVIG: Intravenous immunoglobulin
KDIGO: Kidney Disease: Improving Global Outcomes
LDH: Lactate dehydrogenase
MAHA: Microangiopathic haemolytic anaemia
MAP: Mean arterial pressure
PE: Plasma exchange
PUMCH: Peking Union Medical College Hospital
R-CBA: Residual collagen binding activity
SLE: Systemic lupus erythematosus
SLEDAI: Systemic Lupus Erythematosus Disease Activity Index
SLE-TTP: Systemic lupus erythematosus-associated thrombotic thrombocytopenic purpura
TMA: Thrombotic microangiopathy
TTP: Thrombotic thrombocytopenic purpura
UCTD: Undifferentiated connective tissue disease
